# Crystal structure of a new hybrid anti­mony–halide-based compound for possible non-linear optical applications

**DOI:** 10.1107/S2056989015007379

**Published:** 2015-04-18

**Authors:** Tarek Ben Rhaiem, Habib Boughzala

**Affiliations:** a Laboratoire de Matériaux et Cristallochimie, Faculté des Sciences de Tunis, Université de Tunis El Manar, 2092 Manar II Tunis, Tunisia

**Keywords:** crystal structure, chlorido­anti­monate(III), one-dimensional hybrid compound, (dabcoH_2_)^2+^ cation

## Abstract

The hybrid title compound, *catena*-poly[[[bis­(1,4-diazo­niabi­cyclo­[2.2.2]octa­ne) [tetra­achlorido­anti­monate(III)]-μ-chlorido-[tetra­chlorido­anti­monate(III)]-μ-chlorido]] monohydrate], {(C_6_H_14_N_2_)_2_[Sb_2_Cl_10_]·H_2_O}_*n*_, is self-assembled into alternating organic and inorganic layers parallel to the *bc* plane. The anionic inorganic layer consists of infinite zigzag chains of corner-sharing [SbCl_6_]^3−^ octa­hedra running along the *b* axis. The organic part is made up of 1,4-diazo­niabi­cyclo­[2.2.2]octane dications (dabcoH_2_^2+^). The water mol­ecules in the structure connect inorganic and organic layers. Hydrogen-bonding inter­actions between the ammonium groups, water mol­ecules and Cl atoms ensure the structure cohesion.

## Abstract 2

In a novel one-dimensional hybrid compound, the inorganic part contains zigzag chains formed by corner-sharing [SbCl_6_]^3−^ octa­hedra. 1,4-Diazo­niabi­cyclo­[2.2.2]octane dications cations are lodged around the anionic framework. Water mol­ecules play an important role in the structure connectivity.

## Chemical context   

Organic–inorganic hybrid structures with the general formula {(*R*_a_)^n+^ *M*_b_ *X*_3b+na_} (where *R* is an organic cation; *M* is any trivalent metal and *X* is Cl, Br or/and I) are able to combine desirable characteristics from both types of constituents into a mol­ecular scale composite. These hybrids have been extensively studied for their excitonic and magneto-optical properties. In recent years, a significant number of organic–inorganic hybrid materials based on anti­mony–halide units have been studied. Six-coordinate anti­mony halides can arrange themselves in three-, two- or one-dimensional networks through sharing halides in the Sb*X*_6_ octa­hedra, separated by organic cations (Ben Rhaiem *et al.*, 2013 ▸; Leblanc *et al.*, 2012 ▸; Piecha *et al.*, 2012 ▸; Bujack & Angel, 2005 ▸, 2006 ▸; Bujack & Zaleski, 2004 ▸). One-dimensional extended chains can be formed by one, two or three bridging halides and combinations thereof. The use of one bridging halide leads to two types of chains; if the two bridging halides connecting the central octa­hedron to its neighbours are related *cis*, a zigzag pattern is obtained; if they are *trans*, the chain is linear.
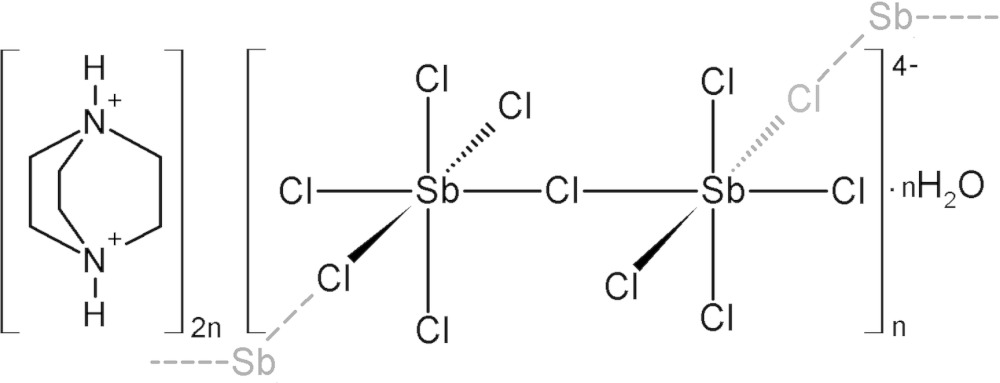


## Structural commentary   

**Figure 1:**
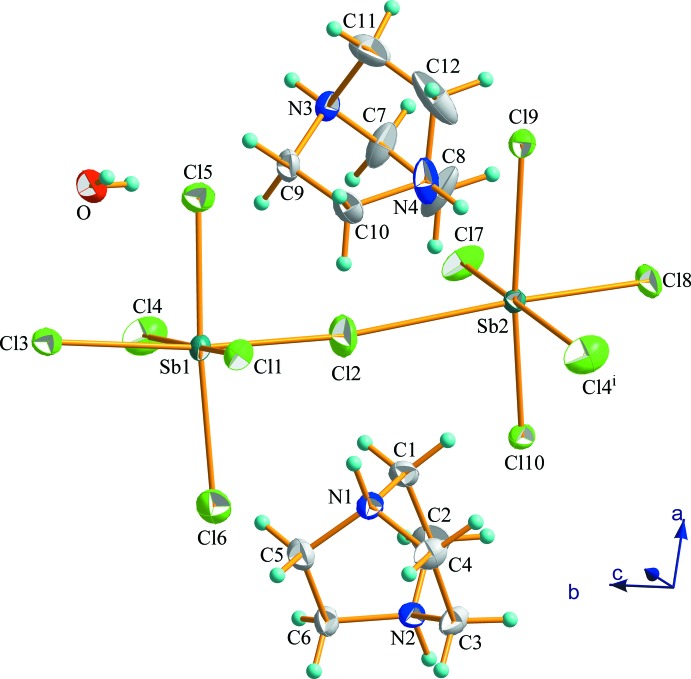
The asymmetric unit of (I) completed by Cl4^i^, showing the atomic numbering scheme. Displacement ellipsoids are shown at 30% probability level. [Symmetry code: (i) *x*, *y* − 1, *z*.]

The asymmetric unit of the new chlorido­anti­monate(III) compound, (C_6_H_14_N_2_)_2_[Sb_2_Cl_10_]·H_2_O, (I), consists of two symmetry-independant (dabcoH_2_)^2+^ dications, a corner sharing bi-octa­hedron deca­chlorido­dianti­monate(III) anion and one crystallization water mol­ecule. The cations are labeled Cat1 (containing atoms N1 and N2) and Cat2 (containing N3 and N4) and the atomic numbering scheme is shown in Fig. 1 ▸.

**Figure 2:**
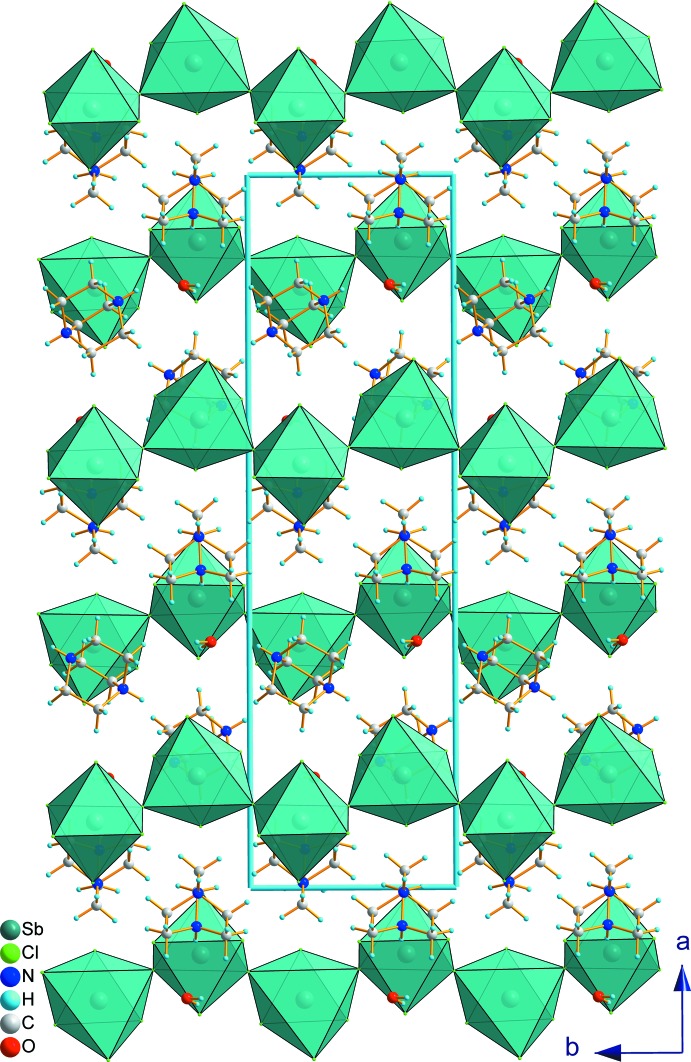
The organic–inorganic layered structure of (I), projected along the *c* axis, showing the zigzag chains of corner-sharing [SbCl_6_]^3−^ octa­hedra.

The structure of the title compound, (I), is self-assembled into an alternating organic and inorganic layered structure. The anionic layer consists of infinite zigzag chains of corner-sharing [SbCl_6_]^3−^ octa­hedra running along the *b* axis. Thus, (I) can be classified among the one-dimensional hybrid structures. The organic part is made up of (dabcoH_2_)^2+^ cations located in the holes around the corner-sharing octa­hedra. The layers are stacked along the *a* axis and water mol­ecules connect the organic and inorganic components (Fig. 2 ▸).

**Figure 3:**
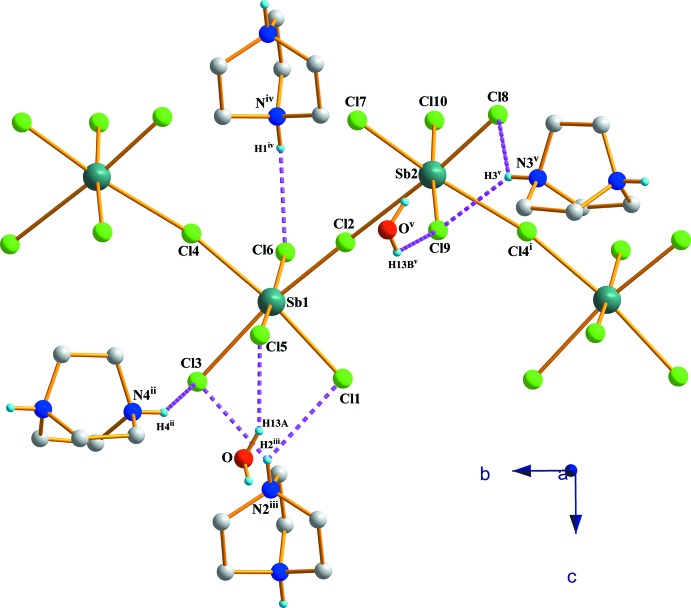
A magnified view of the hydrogen bonding of the inorganic chain in (I). H atoms not involved in hydrogen bonding have been omitted for clarity. [Symmetry codes: (i) *x*, *y* − 1, *z*; (ii) *x*, *y* + 1, *z*; (iii) −*x* + 1, −*y* + 1, *z* + 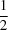; (iv) −*x* + 1, −*y* + 1, *z* − 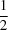; (v) −*x* + 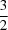, *y* − 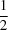, *z* − 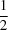.]

The inorganic structural unit part of (I) is build up by two Sb atoms in an octa­hedral coordination ([Sb1Cl_6_]^3−^ and [Sb2Cl_6_]^3−^) joined by the Cl2 ion. Both octa­hedra are severely distorted with Sb—Cl bond lengths lying in the range of 2.5233 (18)–3.073 (2) Å for the bridging ones and 2.4277 (15)–2.8233 (17) Å for the terminal ones. The two bridging halides (Cl2 and Cl4) connecting the central octa­hedron to its neighbours are related *cis*, leading to zigzag chain of corner-sharing [SbCl_6_]^3−^ octa­hedra running along the *b* axis (Fig. 3 ▸).

It is worth noting that at room temperature the DABCO mol­ecule crystallizes in the hexa­gonal system (*P*6_3_/*m*) (Nimmo & Lucas, 1976 ▸). In our case, Cat2 seems to be more distorted than Cat1. In fact, the highest absolute value of the N—C—C—N torsion angle of 7.80 (14)° proves that both (dabcoH_2_)^2+^ cations exhibit deviations from ideal *D*_3*h*_ symmetry. The observed lowering symmetry (hexa­gonal to ortho­rhom­bic) is probably due to the distortion of the (dabcoH_2_)^2+^ cation and can be related to the complex hydrogen-bond network linking the mol­ecular components (cations, anions and water mol­ecules).

The studied compound crystals are transparent and the structure is noncentrosymmetric (*Pna*2_1_). These are two indispensable conditions making this phase a potential promising candidate for non-linear optical (NLO) behaviour as is the case for the well-known KTiOPO_4_ (KTP) and equivalent efficient NLO materials.

## Supra­molecular features   

As shown in Fig. 3 ▸, every bi-octa­hedron unit is linked to four (dabcoH_2_)^2+^ cations and two water mol­ecules *via* hydrogen bonds (Table 1 ▸): on one side Cat1 *via* Cl6⋯H1^iv^—N1^iv^ and Cat 2 by Cl8⋯H3^v^—N3^v^, Cl9⋯H3^v^—N3^v^ [symmetry codes: (iv) −*x* + 1, −*y* + 1, −*z* − 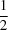; (v) −*x* + 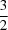, *y* − 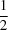, *z* − 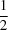] and the other side Cat1 *via* Cl1⋯H2^iii^—N2^iii^, Cl3⋯H2^iii^—N2^iii^ and Cat2 by Cl3⋯H4^ii^—N4^ii^ [symmetry codes: (ii) *x*, *y* + 1, *z*; (iii) −*x* + 1, −*y* + 1, *z* + 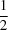]. The water mol­ecules are linked by Cl5⋯H13*A*—O and Cl9⋯H13*B* ^v^—O^v^ [symmetry code: (v) −*x* + 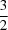, *y* − 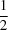, *z* − 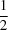].

**Figure 4:**
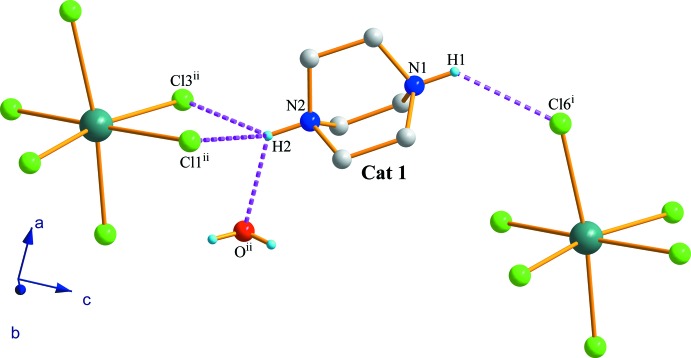
The hydrogen-bonding environment of Cat 1 in (I). Only H atoms involved in hydrogen bonding have been represented. [Symmetry codes: (i) −*x* + 1, −*y* + 1, *z* + 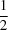; (ii) −*x* + 1, −*y* + 1, *z* − 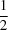.]

**Figure 5:**
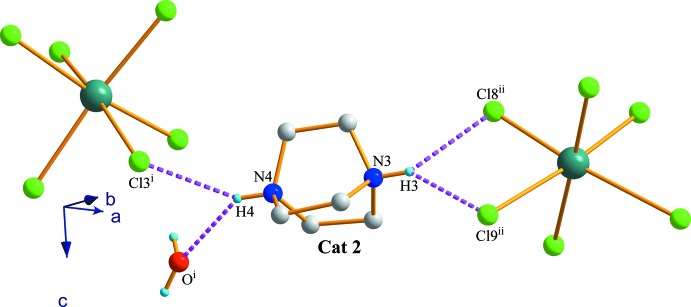
The hydrogen-bonding environment of Cat 2 in (I). Only H atoms involved in hydrogen bonding have been represented. [Symmetry codes: (i) *x*, *y* − 1, *z*; (ii) −*x* + 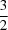, *y* + 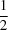, *z* + 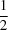.]

Using ammonium groups, both cations (Cat1 and Cat2) are linked to the anionic chains by hydrogen bonds *via* halogenous octa­hedral vertices. As shown in Fig. 4 ▸, Cat1 is linked by N1—H1⋯Cl6^i^ hydrogen bond and three inter­actions between N2—H2 group, both vertices Cl1^ii^—Sb1^ii^, Cl3^ii^—Sb1^ii^ and O atom of the water mol­ecule [symmetry codes: (i) −*x* + 1, −*y* + 1, *z* + 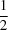; (ii) −*x* + 1, −*y* + 1, *z* − 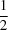]. On the other hand, each ammonium group of Cat 2 inter­acts by two hydrogen bonds. N4—H4 to Cl3^i^—Sb1^i^ and the O atom and N3—H3 group to both Cl8^ii^—Sb2^ii^ and Cl9^ii^—Sb2^ii^ vertices (Fig. 5 ▸) [symmetry codes: (i) *x*, *y* − 1, *z*; (ii) −*x* + 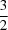, *y* + 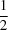, *z* + 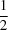].

**Figure 6:**
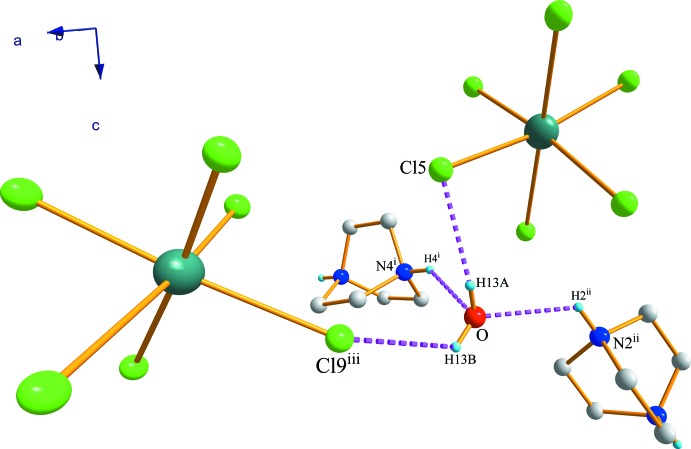
Water-mol­ecule hydrogen-bonding inter­actions in (I). C—H bonds have been omitted for clarity. [Symmetry codes: (i) *x*, *y* + 1, *z*; (ii) −*x* + 1, −*y* + 1, *z* + 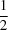; (iii) −*x* + 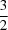, *y* + 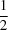, *z* + 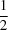.]

As can be seen in Fig. 6 ▸, the water mol­ecule plays an important role in the structure connectivity. It is establishing four hydrogen links joining Cat1 by O⋯H2^ii^—N2^ii^, Cat2 through O⋯H4^i^—N4^i^ and two [SbCl_6_]^3−^ octa­hedra *via* O—H13*A*⋯Cl5 and O—H13*B*⋯Cl9^iii^ [symmetry codes: (i) *x*, *y* + 1, *z*; (ii) −*x* + 1, −*y* + 1, *z* + 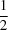; (iii) −*x* + 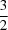, *y* + 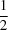, *z* + 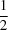].

## Database survey   

A search of the Cambridge Structural Database (Version 5.36; Groom & Allen, 2014 ▸) gave 184 hits for organic–inorganic hybrid materials based on anti­mony chloride units. For this class of compounds with (dabcoH_2_)^2+^ cations, there is only one zero-dimensional compound, (C_6_H_14_N_2_)_2_[Sb_2_Cl_10_]·2H_2_O containing isolated [Sb_2_Cl_10_]^4−^ double octa­hedra, (dabcoH_2_)^2+^ cations and water mol­ecules (Ben Rhaiem *et al.*, 2013 ▸). Indeed, this compound is a pseudo-polymorph over the title compound. For similar one-dimensional compounds with *N*,*N*-di­methyl­ethylenedi­ammonium cations, [(CH_3_)_2_NH(CH_2_)_2_NH_3_]^2+^, see: Bujack & Angel (2006 ▸). For two-dimensional compounds with [{Sb_2_Cl_9_}_*n*_]^3*n*−^ polyanionic layers, see: Bujack & Angel (2005 ▸); Bujack & Zaleski (2004 ▸).

## Synthesis and crystallization   

A mixture of SbCl_3_ (1 mmol) and DABCO (0.5 mmol) was dissolved in a hydro­chloric aqueous solution and stirred for several minutes at 353 K. Colourless crystals suitable for X-ray diffraction analysis were obtained by slow evaporation at room temperature after two weeks.

## Refinement   

Data collection and structure refinement details are summarized in Table 2 ▸. H atoms were localized from geometrical constraint conditions using adequate AFIX and DFIX *SHELXL* (Sheldrick, 2008 ▸) options and parameters were refined with a common isotropic displacement parameter. Water H atoms were found in difference Fourier maps and O—H distances were refined using DFIX and DANG soft restraints. The Flack parameter was refined despite the low Friedel pair coverage because the structure contains a sufficient number of relatively strong anomalous scatterers.

## Supplementary Material

Crystal structure: contains datablock(s) I, New_Global_Publ_Block. DOI: 10.1107/S2056989015007379/vn2091sup1.cif

Structure factors: contains datablock(s) I. DOI: 10.1107/S2056989015007379/vn2091Isup2.hkl

CCDC reference: 943047

Additional supporting information:  crystallographic information; 3D view; checkCIF report

## Figures and Tables

**Table 1:** Hydrogen-bond geometry (, )

*D*H*A*	*D*H	H*A*	*D* *A*	*D*H*A*
N1H1Cl6^i^	0.95	2.67	3.391(6)	134
N2H2Cl1^ii^	0.88	2.78	3.378(4)	126
N2H2Cl3^ii^	0.88	2.62	3.281(6)	133
N2H2O^ii^	0.88	2.46	3.040(7)	124
N3H3Cl8^iii^	0.89	2.82	3.418(7)	126
N3H3Cl9^iii^	0.89	2.38	3.132(9)	143
N4H4Cl3^iv^	0.87	2.66	3.303(6)	131
N4H4O^iv^	0.87	2.30	3.026(8)	143
OH13*A*Cl5	0.84	2.43	3.185(7)	151
OH13*B*Cl9^iii^	0.83	2.66	3.210(5)	126

Symmetry codes: (i) 
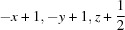
; (ii) 
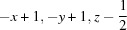
; (iii) 
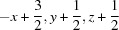
; (iv) 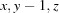.

**Table 2:** Experimental details

Crystal data	
Chemical formula	(C_6_H_14_N_2_)_2_[Sb_2_Cl_10_]H_2_O
*M*_r_	844.40
Crystal system, space group	Orthorhombic, *P* *n* *a*2_1_
Temperature (K)	298
*a*, *b*, *c* ()	29.122(3), 8.4029(10), 11.358(2)
*V* (^3^)	2779.4(7)
*Z*	4
Radiation type	Mo *K*
(mm^1^)	2.92
Crystal size (mm)	0.13 0.06 0.02

Data collection	
Diffractometer	EnrafNonius CAD-4
Absorption correction	scan (North *et al.*, 1968 ▸)
*T*_min_, *T*_max_	0.358, 0.555
No. of measured, independent and observed [*I* > 2(*I*)] reflections	7000, 3492, 2988
*R*_int_	0.041
(sin /)_max_ (^1^)	0.638

Refinement	
*R*[*F* ^2^ > 2(*F* ^2^)], *wR*(*F* ^2^), *S*	0.026, 0.070, 1.09
No. of reflections	3492
No. of parameters	273
No. of restraints	5
H-atom treatment	H atoms treated by a mixture of independent and constrained refinement
_max_, _min_ (e ^3^)	0.72, 0.62
Absolute structure	Flack (1983 ▸), 66 Friedel pairs
Absolute structure parameter	0.01(3)

Computer programs: *CAD-4 EXPRESS* (Duisenberg, 1992 ▸), *XCAD4* (Harms Wocadlo, 1995 ▸), *SHELXS97* and *SHELXL97* (Sheldrick, 2008 ▸), *DIAMOND* (Brandenburg, 2006 ▸) and *publCIF* (Westrip, 2010 ▸).

## supplementary crystallographic information

### Crystal data

**Table:**

(C_6_H_14_N_2_)_2_[Sb_2_Cl_10_]·H_2_O	*D*_x_ = 2.018 Mg m^−^^3^
*M**_r_* = 844.40	Melting point: 594 K
Orthorhombic, *P**n**a*2_1_	Mo *K*α radiation, λ = 0.71073 Å
Hall symbol: P 2c -2n	Cell parameters from 3492 reflections
*a* = 29.122 (3) Å	θ = 2.4–27.0°
*b* = 8.4029 (10) Å	µ = 2.92 mm^−^^1^
*c* = 11.358 (2) Å	*T* = 298 K
*V* = 2779.4 (7) Å^3^	Prism, colourless
*Z* = 4	0.13 × 0.06 × 0.02 mm
*F*(000) = 1640	

### Data collection

**Table:**

Enraf–Nonius CAD-4 diffractometer	2988 reflections with *I* > 2σ(*I*)
Radiation source: fine-focus sealed tube	*R*_int_ = 0.041
Graphite monochromator	θ_max_ = 27.0°, θ_min_ = 2.3°
non–profiled ω/2θ scans	*h* = −37→1
Absorption correction: ψ scan (North *et al.*, 1968)	*k* = −10→10
*T*_min_ = 0.358, *T*_max_ = 0.555	*l* = −1→14
7000 measured reflections	2 standard reflections every 120 min
3492 independent reflections	intensity decay: −1%

### Refinement

**Table:**

Refinement on *F*^2^	Hydrogen site location: inferred from neighbouring sites
Least-squares matrix: full	H atoms treated by a mixture of independent and constrained refinement
*R*[*F*^2^ > 2σ(*F*^2^)] = 0.026	*w* = 1/[σ^2^(*F*_o_^2^) + (0.0316*P*)^2^ + 2.3277*P*] where *P* = (*F*_o_^2^ + 2*F*_c_^2^)/3
*wR*(*F*^2^) = 0.070	(Δ/σ)_max_ = 0.001
*S* = 1.09	Δρ_max_ = 0.72 e Å^−^^3^
3492 reflections	Δρ_min_ = −0.62 e Å^−^^3^
273 parameters	Extinction correction: *SHELXL97* (Sheldrick, 2008), Fc^*^=kFc[1+0.001xFc^2^λ^3^/sin(2θ)]^-1/4^
5 restraints	Extinction coefficient: 0.00168 (12)
Primary atom site location: structure-invariant direct methods	Absolute structure: Flack (1983), 66 Friedel pairs
Secondary atom site location: difference Fourier map	Absolute structure parameter: −0.01 (3)

### Special details

**Table:**

Experimental. Absorption correction: North *et al.* (1968) Number of psi-scan sets used was 6 Theta correction was applied. Averaged transmission function was used. No Fourier smoothing was applied.
Geometry. All esds (except the esd in the dihedral angle between two l.s. planes) are estimated using the full covariance matrix. The cell esds are taken into account individually in the estimation of esds in distances, angles and torsion angles; correlations between esds in cell parameters are only used when they are defined by crystal symmetry. An approximate (isotropic) treatment of cell esds is used for estimating esds involving l.s. planes.
Refinement. Refinement of F^2^ against ALL reflections. The weighted R-factor wR and goodness of fit S are based on F^2^, conventional R-factors R are based on F, with F set to zero for negative F^2^. The threshold expression of F^2^ > 2sigma(F^2^) is used only for calculating R-factors(gt) etc. and is not relevant to the choice of reflections for refinement. R-factors based on F^2^ are statistically about twice as large as those based on F, and R- factors based on ALL data will be even larger.

### Fractional atomic coordinates and isotropic or equivalent isotropic displacement parameters (Å^2^)

**Table:**

	*x*	*y*	*z*	*U*_iso_*/*U*_eq_	
Sb1	0.593657 (12)	0.74481 (3)	0.49688 (3)	0.02787 (11)	
Sb2	0.657146 (11)	0.24077 (3)	0.22050 (3)	0.02842 (11)	
Cl1	0.57361 (5)	0.54093 (18)	0.66622 (15)	0.0452 (4)	
Cl2	0.61712 (8)	0.5151 (2)	0.3671 (2)	0.0760 (6)	
Cl3	0.57606 (5)	0.97355 (17)	0.67100 (15)	0.0437 (3)	
Cl4	0.61408 (7)	0.9753 (2)	0.3459 (2)	0.0765 (6)	
Cl5	0.67713 (6)	0.75095 (17)	0.56999 (19)	0.0522 (4)	
Cl6	0.50864 (7)	0.7328 (2)	0.3883 (2)	0.0619 (5)	
Cl7	0.69068 (7)	0.4519 (2)	0.09980 (17)	0.0645 (5)	
Cl8	0.68555 (6)	0.0345 (2)	0.08973 (16)	0.0562 (5)	
Cl9	0.74068 (5)	0.19561 (18)	0.34215 (18)	0.0430 (3)	
Cl10	0.58708 (5)	0.25828 (14)	0.1013 (2)	0.0478 (4)	
N1	0.49311 (19)	0.2444 (5)	0.5900 (5)	0.0390 (12)	
H1	0.5113 (15)	0.2480 (6)	0.659 (6)	0.047*	
N2	0.44602 (17)	0.2354 (5)	0.4084 (5)	0.0378 (11)	
H2	0.4294 (4)	0.2318 (5)	0.3440 (16)	0.045*	
N3	0.72003 (16)	0.3837 (5)	0.7294 (5)	0.0389 (11)	
H3	0.7352 (10)	0.475 (6)	0.7261 (5)	0.047*	
N4	0.6767 (2)	0.1317 (6)	0.7367 (7)	0.068 (2)	
H4	0.6616 (14)	0.044 (8)	0.7384 (7)	0.082*	
C1	0.5235 (2)	0.2498 (6)	0.4859 (7)	0.0455 (16)	
H1A	0.5447	0.1609	0.4882	0.055*	
H1B	0.5412	0.3475	0.4867	0.055*	
C2	0.4952 (2)	0.2417 (8)	0.3748 (8)	0.059 (2)	
H2A	0.5010	0.3347	0.3264	0.071*	
H2B	0.5033	0.1476	0.3299	0.071*	
C3	0.4365 (2)	0.0911 (7)	0.4788 (6)	0.0481 (17)	
H3A	0.4434	−0.0030	0.4326	0.058*	
H3B	0.4043	0.0880	0.5001	0.058*	
C4	0.4656 (2)	0.0921 (7)	0.5887 (6)	0.0479 (15)	
H4A	0.4860	0.0010	0.5891	0.057*	
H4B	0.4461	0.0865	0.6579	0.057*	
C5	0.4622 (2)	0.3851 (8)	0.5871 (6)	0.0510 (16)	
H5A	0.4802	0.4821	0.5888	0.061*	
H5B	0.4423	0.3844	0.6556	0.061*	
C6	0.4333 (2)	0.3800 (6)	0.4754 (6)	0.0467 (17)	
H6A	0.4010	0.3773	0.4953	0.056*	
H6B	0.4391	0.4742	0.4283	0.056*	
C7	0.7097 (3)	0.3302 (8)	0.6095 (6)	0.063 (2)	
H7A	0.7381	0.3127	0.5668	0.075*	
H7B	0.6924	0.4116	0.5685	0.075*	
C8	0.6829 (4)	0.1808 (11)	0.6134 (8)	0.092 (4)	
H8A	0.6532	0.1972	0.5767	0.110*	
H8B	0.6989	0.0981	0.5702	0.110*	
C9	0.6768 (2)	0.4080 (7)	0.7935 (7)	0.0473 (15)	
H9A	0.6577	0.4839	0.7516	0.057*	
H9B	0.6831	0.4505	0.8712	0.057*	
C10	0.6519 (2)	0.2512 (6)	0.8042 (8)	0.0479 (18)	
H10A	0.6502	0.2195	0.8862	0.057*	
H10B	0.6208	0.2617	0.7744	0.057*	
C11	0.7484 (3)	0.2641 (9)	0.7941 (10)	0.081 (3)	
H11A	0.7525	0.2964	0.8754	0.097*	
H11B	0.7784	0.2535	0.7577	0.097*	
C12	0.7221 (3)	0.1056 (10)	0.7878 (11)	0.107 (4)	
H12A	0.7391	0.0303	0.7399	0.129*	
H12B	0.7190	0.0612	0.8662	0.129*	
O	0.65412 (17)	0.8095 (7)	0.8407 (6)	0.0637 (14)	
H13A	0.662 (3)	0.760 (8)	0.780 (4)	0.070*	
H13B	0.674 (2)	0.788 (9)	0.890 (5)	0.070*	

### Atomic displacement parameters (Å^2^)

**Table:**

	*U*^11^	*U*^22^	*U*^33^	*U*^12^	*U*^13^	*U*^23^
Sb1	0.0343 (2)	0.02365 (18)	0.0257 (2)	−0.00135 (12)	0.00135 (16)	−0.00099 (18)
Sb2	0.03007 (18)	0.02876 (19)	0.0264 (2)	0.00021 (12)	−0.00028 (17)	−0.0021 (2)
Cl1	0.0497 (9)	0.0439 (7)	0.0421 (8)	−0.0053 (6)	−0.0007 (7)	−0.0001 (7)
Cl2	0.0915 (15)	0.0703 (11)	0.0662 (14)	0.0140 (11)	0.0062 (12)	−0.0308 (11)
Cl3	0.0418 (8)	0.0419 (7)	0.0473 (8)	0.0013 (6)	−0.0043 (7)	0.0077 (7)
Cl4	0.0833 (14)	0.0808 (13)	0.0655 (14)	−0.0231 (11)	−0.0066 (13)	0.0276 (12)
Cl5	0.0457 (8)	0.0532 (9)	0.0578 (12)	−0.0055 (7)	0.0018 (8)	0.0039 (8)
Cl6	0.0538 (10)	0.0626 (11)	0.0695 (14)	−0.0031 (8)	0.0168 (10)	0.0005 (10)
Cl7	0.0750 (12)	0.0718 (11)	0.0466 (10)	−0.0361 (9)	−0.0079 (10)	0.0184 (9)
Cl8	0.0638 (11)	0.0643 (9)	0.0406 (9)	0.0285 (8)	−0.0105 (8)	−0.0222 (8)
Cl9	0.0429 (7)	0.0501 (7)	0.0360 (7)	0.0012 (7)	0.0007 (7)	−0.0113 (7)
Cl10	0.0447 (8)	0.0333 (7)	0.0652 (12)	0.0041 (5)	−0.0225 (8)	−0.0034 (7)
N1	0.044 (3)	0.038 (3)	0.035 (3)	0.001 (2)	−0.007 (2)	0.000 (2)
N2	0.039 (3)	0.036 (2)	0.038 (3)	0.0019 (18)	−0.007 (2)	0.003 (2)
N3	0.046 (3)	0.033 (2)	0.038 (3)	−0.0052 (18)	0.004 (3)	−0.003 (2)
N4	0.089 (5)	0.028 (2)	0.088 (5)	−0.010 (3)	0.049 (4)	0.000 (3)
C1	0.034 (3)	0.041 (3)	0.062 (5)	−0.004 (2)	0.004 (3)	0.003 (3)
C2	0.045 (4)	0.089 (6)	0.043 (4)	−0.001 (3)	0.003 (3)	−0.001 (4)
C3	0.057 (4)	0.031 (2)	0.057 (4)	−0.008 (2)	−0.021 (4)	0.009 (3)
C4	0.053 (4)	0.046 (3)	0.044 (4)	−0.011 (3)	−0.002 (3)	0.013 (3)
C5	0.055 (4)	0.051 (3)	0.047 (4)	0.012 (3)	0.000 (3)	−0.009 (3)
C6	0.057 (4)	0.032 (3)	0.051 (4)	0.010 (3)	−0.014 (3)	−0.004 (3)
C7	0.110 (7)	0.051 (3)	0.026 (3)	−0.031 (4)	0.009 (4)	−0.004 (3)
C8	0.133 (9)	0.087 (6)	0.054 (5)	−0.071 (6)	0.028 (6)	−0.035 (5)
C9	0.052 (4)	0.038 (3)	0.052 (4)	0.002 (3)	0.008 (3)	−0.015 (3)
C10	0.047 (4)	0.051 (4)	0.046 (4)	0.004 (3)	0.017 (3)	0.004 (3)
C11	0.056 (5)	0.111 (7)	0.075 (7)	0.033 (5)	−0.011 (5)	−0.004 (5)
C12	0.103 (8)	0.071 (5)	0.148 (10)	0.047 (5)	0.079 (7)	0.060 (6)
O	0.055 (3)	0.067 (3)	0.069 (4)	0.017 (2)	−0.010 (3)	−0.016 (3)

### Geometric parameters (Å, º)

**Table:**

Sb1—Cl2	2.5233 (18)	C1—H1B	0.9700
Sb1—Cl5	2.5695 (18)	C2—H2A	0.9700
Sb1—Cl1	2.6411 (17)	C2—H2B	0.9700
Sb1—Cl4	2.654 (2)	C3—C4	1.507 (9)
Sb1—Cl6	2.768 (2)	C3—H3A	0.9700
Sb1—Cl3	2.8051 (17)	C3—H3B	0.9700
Sb2—Cl8	2.4277 (15)	C4—H4A	0.9700
Sb2—Cl7	2.4457 (17)	C4—H4B	0.9700
Sb2—Cl10	2.4532 (16)	C5—C6	1.521 (9)
Sb2—Cl9	2.8233 (17)	C5—H5A	0.9700
Sb2—Cl2	3.073 (2)	C5—H5B	0.9700
Sb2—Cl4^i^	2.9291 (19)	C6—H6A	0.9700
N1—C1	1.477 (9)	C6—H6B	0.9700
N1—C5	1.487 (8)	C7—C8	1.480 (10)
N1—C4	1.510 (7)	C7—H7A	0.9700
N1—H1	0.9496	C7—H7B	0.9700
N2—C3	1.478 (7)	C8—H8A	0.9700
N2—C6	1.481 (7)	C8—H8B	0.9700
N2—C2	1.483 (9)	C9—C10	1.509 (7)
N2—H2	0.8784	C9—H9A	0.9700
N3—C7	1.465 (8)	C9—H9B	0.9700
N3—C9	1.469 (8)	C10—H10A	0.9700
N3—C11	1.494 (9)	C10—H10B	0.9700
N3—H3	0.8858	C11—C12	1.538 (11)
N4—C10	1.456 (8)	C11—H11A	0.9700
N4—C12	1.462 (12)	C11—H11B	0.9700
N4—C8	1.471 (11)	C12—H12A	0.9700
N4—H4	0.8565	C12—H12B	0.9700
C1—C2	1.508 (11)	O—H13A	0.84 (2)
C1—H1A	0.9700	O—H13B	0.82 (2)
			
Cl2—Sb1—Cl5	87.02 (7)	C1—C2—H2B	110.0
Cl2—Sb1—Cl1	89.38 (7)	H2A—C2—H2B	108.4
Cl5—Sb1—Cl1	89.25 (6)	N2—C3—C4	109.8 (5)
Cl2—Sb1—Cl4	96.90 (9)	N2—C3—H3A	109.7
Cl5—Sb1—Cl4	88.98 (6)	C4—C3—H3A	109.7
Cl1—Sb1—Cl4	173.38 (6)	N2—C3—H3B	109.7
Cl2—Sb1—Cl6	87.36 (7)	C4—C3—H3B	109.7
Cl5—Sb1—Cl6	172.33 (7)	H3A—C3—H3B	108.2
Cl1—Sb1—Cl6	95.92 (6)	C3—C4—N1	108.1 (5)
Cl4—Sb1—Cl6	86.51 (6)	C3—C4—H4A	110.1
Cl2—Sb1—Cl3	170.28 (7)	N1—C4—H4A	110.1
Cl5—Sb1—Cl3	86.05 (5)	C3—C4—H4B	110.1
Cl1—Sb1—Cl3	83.72 (5)	N1—C4—H4B	110.1
Cl4—Sb1—Cl3	89.79 (6)	H4A—C4—H4B	108.4
Cl6—Sb1—Cl3	100.12 (5)	N1—C5—C6	109.3 (5)
Cl8—Sb2—Cl7	92.23 (8)	N1—C5—H5A	109.8
Cl8—Sb2—Cl10	89.35 (6)	C6—C5—H5A	109.8
Cl7—Sb2—Cl10	88.82 (7)	N1—C5—H5B	109.8
Cl8—Sb2—Cl9	84.83 (5)	C6—C5—H5B	109.8
Cl7—Sb2—Cl9	91.59 (6)	H5A—C5—H5B	108.3
Cl10—Sb2—Cl9	174.17 (6)	N2—C6—C5	108.3 (5)
Cl4^i^—Sb2—Cl10	87.62 (5)	N2—C6—H6A	110.0
Cl2—Sb2—Cl10	86.49 (6)	C5—C6—H6A	110.0
Cl4^i^—Sb2—Cl9	91.63 (5)	N2—C6—H6B	110.0
Cl2—Sb2—Cl9	99.35 (5)	C5—C6—H6B	110.0
Cl8—Sb2—Cl4^i^	84.24 (6)	H6A—C6—H6B	108.4
Cl4^i^—Sb2—Cl2	98.36 (5)	N3—C7—C8	109.9 (6)
Cl2—Sb2—Cl7	84.89 (6)	N3—C7—H7A	109.7
Cl2—Sb2—Cl8	174.98 (6)	C8—C7—H7A	109.7
Cl4^i^—Sb2—Cl7	175.00 (6)	N3—C7—H7B	109.7
C1—N1—C5	108.7 (5)	C8—C7—H7B	109.7
C1—N1—C4	109.6 (5)	H7A—C7—H7B	108.2
C5—N1—C4	110.6 (5)	N4—C8—C7	109.3 (6)
C1—N1—H1	109.3	N4—C8—H8A	109.8
C5—N1—H1	109.3	C7—C8—H8A	109.8
C4—N1—H1	109.3	N4—C8—H8B	109.8
C3—N2—C6	110.4 (5)	C7—C8—H8B	109.8
C3—N2—C2	110.4 (5)	H8A—C8—H8B	108.3
C6—N2—C2	110.1 (5)	N3—C9—C10	109.3 (5)
C3—N2—H2	108.6	N3—C9—H9A	109.8
C6—N2—H2	108.6	C10—C9—H9A	109.8
C2—N2—H2	108.6	N3—C9—H9B	109.8
C7—N3—C9	109.1 (5)	C10—C9—H9B	109.8
C7—N3—C11	111.4 (6)	H9A—C9—H9B	108.3
C9—N3—C11	108.9 (6)	N4—C10—C9	108.7 (5)
C7—N3—H3	109.1	N4—C10—H10A	109.9
C9—N3—H3	109.1	C9—C10—H10A	109.9
C11—N3—H3	109.1	N4—C10—H10B	109.9
C10—N4—C12	110.1 (7)	C9—C10—H10B	109.9
C10—N4—C8	111.6 (7)	H10A—C10—H10B	108.3
C12—N4—C8	108.0 (8)	N3—C11—C12	106.5 (7)
C10—N4—H4	109.0	N3—C11—H11A	110.4
C12—N4—H4	109.0	C12—C11—H11A	110.4
C8—N4—H4	109.0	N3—C11—H11B	110.4
N1—C1—C2	110.0 (5)	C12—C11—H11B	110.4
N1—C1—H1A	109.7	H11A—C11—H11B	108.6
C2—C1—H1A	109.7	N4—C12—C11	109.8 (6)
N1—C1—H1B	109.7	N4—C12—H12A	109.7
C2—C1—H1B	109.7	C11—C12—H12A	109.7
H1A—C1—H1B	108.2	N4—C12—H12B	109.7
N2—C2—C1	108.3 (6)	C11—C12—H12B	109.7
N2—C2—H2A	110.0	H12A—C12—H12B	108.2
C1—C2—H2A	110.0	H13A—O—H13B	105 (4)
N2—C2—H2B	110.0		

Symmetry code: (i) *x*, *y*−1, *z*.

### Hydrogen-bond geometry (Å, º)

**Table:**

*D*—H···*A*	*D*—H	H···*A*	*D*···*A*	*D*—H···*A*
N1—H1···Cl6^ii^	0.95	2.67	3.391 (6)	134
N2—H2···Cl1^iii^	0.88	2.78	3.378 (4)	126
N2—H2···Cl3^iii^	0.88	2.62	3.281 (6)	133
N2—H2···O^iii^	0.88	2.46	3.040 (7)	124
N3—H3···Cl8^iv^	0.89	2.82	3.418 (7)	126
N3—H3···Cl9^iv^	0.89	2.38	3.132 (9)	143
N4—H4···Cl3^i^	0.87	2.66	3.303 (6)	131
N4—H4···O^i^	0.87	2.30	3.026 (8)	143
O—H13*A*···Cl5	0.84	2.43	3.185 (7)	151
O—H13*B*···Cl9^iv^	0.83	2.66	3.210 (5)	126

Symmetry codes: (i) *x*, *y*−1, *z*; (ii) −*x*+1, −*y*+1, *z*+1/2; (iii) −*x*+1, −*y*+1, *z*−1/2; (iv) −*x*+3/2, *y*+1/2, *z*+1/2.
